# Patient perception of anticoagulant treatment for stroke prevention (RE-SONANCE study)

**DOI:** 10.1136/openhrt-2019-001202

**Published:** 2020-03-24

**Authors:** Dragos Vinereanu, Dmitry Napalkov, Jutta Bergler-Klein, Bela Benczur, Martin Ciernik, Nina Gotcheva, Alexey Medvedchikov, Pentti Põder, Dragan Simic, Andris Skride, Wenbo Tang, Maria Trusz-Gluza, Jiri Vesely, Tatiana Vishnepolsky, Mirej Vrabec

**Affiliations:** 1 Cardiology, University and Emergency Hospital of Bucharest, Bucharest, Other, Romania; 2 Department of Internal Medicine, I.M. Sechenov First Moscow State Medical University (Sechenov University), Moscow, Russian Federation; 3 Department of Cardiology, University Clinic of Internal Medicine II, Medical University of Vienna, Vienna, Austria; 4 1st Department of Internal Medicine (Cardiology/Nephrology), Tolna County ‘Balassa Janos’ Teaching Hospital, Szekszard, Hungary; 5 Boehringer Ingelheim RCV GmbH & Co. KG, Vienna, Austria; 6 Department of Cardiology, National Cardiology Hospital, Sofia, Bulgaria; 7 Department of Cardiology, North Estonia Medical Centre Foundation, Tallinn, Estonia; 8 Department of Cardiology, Clinical Centre of Serbia, Belgrade, Serbia; 9 Cardiology Department, Riga Stradins University, Riga, Latvia; 10 Biostatistics and Data Sciences, Boehringer Ingelheim Pharmaceuticals, Inc, Ridgefield, Connecticut, USA; 11 First Department of Cardiology, Silesian Medical University, Katowice, Poland; 12 Faculty of Medicine in Hradec Kralové, Charles University, Broumov, Czech Republic; 13 Clalit Health Services, Bonen Clinic, Haifa, Israel; 14 Department of Cardiology, General Hospital Celje, Celje, Slovenia

**Keywords:** stroke, atrial fibrillation, quality of care and outcomes

## Abstract

**Objective:**

We evaluated atrial fibrillation (AF) patients’ perceptions of anticoagulation treatment with dabigatran or a vitamin K antagonist (VKA) for stroke prevention, according to accepted indications.

**Methods:**

The RE-SONANCE observational, prospective, multicentre, international study used the validated Perception on Anticoagulant Treatment Questionnaire (PACT-Q) to assess patients with AF already taking a VKA who were switched to dabigatran (cohort A), and newly diagnosed patients initiated on either dabigatran or a VKA (cohort B). Visit 1 (V1) was at baseline, and visit 2 (V2) and visit 3 (V3) were at 30–45 and 150–210 days after baseline, respectively. Primary outcomes were treatment satisfaction and convenience in cohort A at V2 and V3 versus baseline, and in cohort B for dabigatran and a VKA at V2 and V3.

**Results:**

The main analysis set comprised 4100 patients in cohort A and 5365 in cohort B (dabigatran: 3179; VKA: 2186). In cohort A, PACT-Q2 improved significantly (p<0.001 for all) for treatment convenience (mean change V1 vs V2=20.72; SD=21.50; V1 vs V3=24.54; SD=22.85) and treatment satisfaction (mean change V1 vs V2=17.60; SD=18.76; V1 vs V3=21.04; SD=20.24). In cohort B, mean PACT-Q2 scores at V2 and V3 were significantly higher (p<0.001 for all) for dabigatran versus a VKA for treatment convenience (V2=18.38; SE =0.51; V3=23.34; SE=0.51) and satisfaction (V2=15.88; SE=0.39; V3=19.01; SE=0.41).

**Conclusions:**

Switching to dabigatran from long-term VKA therapy or newly initiated dabigatran is associated with improved patient treatment convenience and satisfaction compared with VKA therapy.

**Key questions**

**What is already known about this subject?**

- Including patients in their anticoagulant treatment decision-making is important, and using educational intervention programmes targeting both patients and physicians can improve the use of oral anticoagulation in patients with atrial fibrillation (AF) who are at risk of stroke. Evaluating patients’ perspectives of anticoagulation treatment satisfaction is also important, as this may impact adherence and therefore outcomes. Nevertheless, data on patients’ perception of long-term anticoagulation therapy in non-valvular AF (NVAF) are limited.

**What does this study add?**

- The RE-SONANCE observational prospective study evaluated the perceptions of anticoagulation treatment and treatment convenience of patients with NVAF treated with dabigatran or a vitamin K antagonist (VKA) for stroke prevention, according to accepted indications.

**How might this impact on clinical practice?**

- Switching to dabigatran from long-term VKA therapy or newly initiated dabigatran is associated with improved patient treatment convenience and satisfaction compared with VKA therapy. This may be important in the prevention of stroke and systemic embolism in patients with AF patients at moderate-to-high risk.

## Introduction

Prior to the availability of novel oral anticoagulants (NOACs), vitamin K antagonists (VKAs) such as warfarin were the mainstay of anticoagulation therapy for prevention of stroke in non-valvular atrial fibrillation (NVAF). However, there is a real-world underuse of oral anticoagulants (OACs) in patients with atrial fibrillation (AF) who should be receiving treatment, resulting in a great number of preventable ischaemic strokes in these undertreated patients.^1^ Several factors contributed to suboptimal adherence with VKAs, including the narrow therapeutic window of VKAs, numerous food and drug interactions, a variable dose–response relationship and the requirement for frequent anticoagulation monitoring and dose adjustment with VKAs. The NOACs circumvent many of these problems and are currently recommended by European guidelines as the preferred anticoagulation treatment in patients with AF at risk of stroke.^2–4^

Including patients in their treatment decision-making is important,^3^ and using educational intervention programmes targeting both patients and physicians can improve the use of OACs in patients with AF at risk of stroke.^5–9^ Evaluating patients’ perspectives of anticoagulation treatment satisfaction is also important, as this may impact adherence and therefore outcomes.^10^ However, data on patients’ perception of long-term anticoagulation therapy in NVAF are limited.^5 11–13^

Therefore, we have evaluated perceptions of anticoagulation treatment and treatment convenience of patients with NVAF treated with dabigatran or a VKA for stroke prevention, according to accepted indications. The RE-SONANCE observational prospective study (NCT02684981), conducted in 11 European countries and Israel, used the validated Perception on Anticoagulant Treatment Questionnaire (PACT-Q)^10 14^ to assess two different patient cohorts: patients with NVAF taking a VKA who were switched to dabigatran and newly diagnosed patients with NVAF initiated on either dabigatran or a VKA.

## Methods

### Study population and trial design

The study was conducted in Austria, Bulgaria, Czech Republic, Estonia, Hungary, Israel, Latvia, Poland, Romania, Russian Federation, Serbia and Slovenia. Eligible patients were ≥18 years old with NVAF and an indication for anticoagulation therapy for stroke prevention, not participating in any other clinical trial (for a drug or device) or registry. Cohort A included patients with NVAF switched from a VKA to dabigatran (≥3 months continuous VKA treatment for stroke prevention prior to baseline), while cohort B included newly diagnosed patients with NVAF initiated on either dabigatran or a VKA (with no use of any OAC within 1 year prior to enrolment).

### Assessments

Collection of patient characteristics and treatment data was managed during routine clinic visits at three recommended time points. Visit 1 (V1) was at baseline, when patients were either switched from a VKA to dabigatran or started on dabigatran or VKA treatment. Visit 2 (V2) was 30–45 days after baseline. Visit 3 (V3) was 150–210 days after baseline. While these proposed time periods were provided as guidance to the treating physician, the timing of each visit was based on real-life practice in the respective countries. Therefore, visits were also performed by sites outside these windows, so the time points were revised and extended to: V2, 7–124 days after baseline and V3, 125–365 days after baseline. PACT-Q was used as a self-administered questionnaire.^10 14^ Patient expectations regarding their anticoagulant treatment were assessed using PACT-Q1. PACT-Q2 assessed perceptions regarding convenience, anticoagulant treatment satisfaction, burden of disease and treatment. Cohort A completed PACT-Q2 at baseline, V2 and V3, and cohort B completed PACT-Q1 at baseline and PACT-Q2 at V2 and V3.

### Outcomes

#### Primary outcomes

The primary outcome for cohort A was patient satisfaction with anticoagulant treatment and treatment convenience (PACT-Q2 domains) at V2 and V3 versus baseline. For cohort B, it was patient satisfaction with anticoagulant treatment and treatment convenience (PACT-Q2 domains) between treatment groups (dabigatran vs VKA) at V2 and V3.

#### Secondary outcomes

For cohort A, this was to evaluate the mean changes over time in patients' satisfaction and treatment convenience (PACT-Q2 domain) between V3 and V2; for cohort B, it was anticoagulation treatment expectations (PACT-Q1 items) at baseline.

#### Safety outcomes

Safety was evaluated separately for patients in cohorts A and B with follow-up for potential adverse drug reactions (ADRs) and fatal adverse events.

### Statistical analyses

#### Sample size and analysis sets

The planned sample size was 9000 patients. For cohort A (assuming a two-sided alpha of 0.05 and that 20% of patients would be lost to follow-up), a total sample size of 3000 patients would provide >80% power to detect a standardised mean difference of 0.06 in PACT-Q2 scores between two assessments. For cohort B (assuming a two-sided alpha of 0.05, a 1:1 ratio of dabigatran and VKA patients and a 30% loss to follow-up and matching), a total sample size of 6000 patients would provide >80% power to detect a standardised mean difference of 0.065 in PACT-Q2 scores between dabigatran and a VKA at each assessment.

Enrolled patients were those who met all eligibility criteria. The main analysis set (MAS) comprised all eligible patients with known treatment; the safety analysis set comprised all enrolled patients with follow-up data. The propensity score matched set (PSMS) compared PACT-Q2 treatment differences between dabigatran and a VKA in cohort B; all patients matched with a 1:n (n, range 1–3) ratio (VKA:dabigatran) based on propensity scores calculated using a logistic regression model. Patients who permanently discontinued initial anticoagulation treatment at the time of an assessment were excluded from all analyses where data from that assessment were included.

#### Assessment of PACT-Q2 scores

For cohort A, mean differences in PACT-Q2 scores between visits were assessed using paired t-tests. In cohort B, mean differences in PACT-Q2 scores between treatment groups were assessed using the PSMS and the random intercept model. Paired t-tests were used for sensitivity analysis. The baseline variables used in the PSMS included: sex (men/women); age (<65/≥65 to <75/≥75 years); reimbursement status (reimbursed/partially reimbursed/private pay/other); physician specialty (cardiologist/internist/neurologist/general practitioner/other); HAS-BLED score (low (<3)/high (≥3) risk); CHA_2_DS_2_-VASc score (low or intermediate (<2)/high (≥2) risk); number of concomitant medications (0/1–3/≥4); type of concomitant medication (prescription/no prescription of (i) antiarrhythmics, (ii) antiplatelets or (iii) non-steroidal anti-inflammatory drugs); number of concomitant therapies (0/≥1); presence of comorbidities (presence/absence of (i) malignancy or (ii) gastro-oesophageal reflux disease or gastroduodenal ulcer disease). The planned primary analysis was originally based on a 1:1 patient ratio. However, unequal enrolment (reflecting real-world treatment patterns and country recruitment) led to an adjustment of this analysis. Therefore, due to the variable size of the matched sets, the primary analysis of PACT-Q2 scores was based on the random intercept model, where a variance in the unequal groups (1:n matching) is compared and one group contains ‘repeated’ observations.

### Assessment of PACT-Q1 scores

PACT-Q1 scores in cohort B at baseline were summarised descriptively for all patients and between treatment groups.

## Results

### Patient disposition

In total, 9472 patients with NVAF were enrolled from 698 sites in 11 European countries and Israel. The flow of patients through the study is summarised in online appendix figure 1. A total of 577 patients (240 (5.9%) in cohort A; 337 in cohort B (210 (6.6%) receiving dabigatran and 127 (5.8%) receiving a VKA)) permanently discontinued treatment during the study or had unknown treatment status at the end of observation. In cohort B, of those patients discontinuing VKA therapy, 42 (1.9%) were switched to dabigatran. All data collected prior to premature discontinuation were included in the analyses.

10.1136/openhrt-2019-001202.supp1 Supplementary data

### Patient characteristics and anticoagulation treatment

Tables 1–3 summarise the demographic and baseline characteristics, healthcare system characteristics and physician-rated risk scores in the MAS. Most patients had a high risk of stroke/bleeding complications, had comorbidities and were receiving concomitant medications. Creatinine clearance (calculated at each visit using the Cockcroft–Gault equation) was stable in both cohorts (data not shown). There was a high risk of stroke or systemic embolism (CHA_2_DS_2_-VASc score ≥2) and bleeding complications (HAS-BLED score ≥3), reported in 88% and 59% of patients in cohort A, 88% and 29% of cohort B dabigatran-treated patients and 91% and 31% of cohort B VKA-treated patients, respectively.

**Table 1:** Baseline demographics, comorbidities and concomitant medications (main analysis set)

	Cohort A (switched from VKA to dabigatran)		Cohort B (newly initiated on dabigatran or a VKA)	
	Total (n=4100)	Dabigatran (n=3179)	VKA (n=2186)	Total (n=5365)
Mean age, years (SD)	70.5 (9.6)	68.6 (10.1)	68.5 (9.5)	68.6 (9.9)
Range, years	18–100	22–95	18–95	18–95
Age, n (%)				
<65 years	1029 (25.1)	1042 (32.8)	723 (33.1)	1765 (32.9)
65 to <75 years	1552 (37.9)	1154 (36.3)	803 (36.7)	1957 (36.5)
≥75 years	1519 (37.0)	983 (30.9)	660 (30.2)	1643 (30.6)
Sex, n (%)				
Female	1998 (48.7)	1602 (50.4)	1080 (49.4)	2682 (50.0)
Male	2102 (51.3)	1577 (49.6)	1106 (50.6)	2683 (50.0)
Mean (SD) weight, kg	84.1 (16.2)	82.8 (15.5)	82.5 (15.0)	82.6 (15.3)
Comorbidities, n (%)	3541 (86.4)	2651 (83.4)	1986 (90.9)	4637 (86.4)
Blood and immune system	62 (1.5)	40 (1.3)	29 (1.3)	69 (1.3)
GI/metabolic	1618 (39.5)	1044 (32.8)	774 (35.4)	1818 (33.9)
Heart and blood vessels	3390 (82.7)	2548 (80.2)	1904 (87.1)	4452 (83.0)
Previous MI	95 (2.3)	77 (2.4)	78 (3.6)	155 (2.9)
CVA/previous TIA	247 (6.0)	168 (5.3)	118 (5.4)	286 (5.3)
Previous CHD	1048 (25.6)	749 (23.6)	625 (28.6)	1374 (25.6)
Hypertension (arterial)	2921 (71.2)	2228 (70.1)	1689 (77.3)	3917 (73.0)
Lung	285 (7.0)	166 (5.2)	131 (6.0)	297 (5.5)
Malignancy	48 (1.2)	28 (0.9)	7 (0.3)	35 (0.7)
Urogenital system	308 (7.5)	217 (6.8)	175 (8.0)	392 (7.3)
Other*	632 (15.4)	416 (13.1)	270 (12.4)	686 (12.8)
Concomitant medications, n (%)	3542 (86.4)	652 (83.4)	1993 (91.2)	4645 (86.6)
Antacids	393 (9.6)	248 (7.8)	167 (7.6)	415 (7.7)
Antidiabetes drugs	687 (16.8)	392 (12.3)	344 (15.7)	736 (13.7)
Antihypertensive drugs	3229 (78.8)	2428 (76.4)	1839 (84.1)	4267 (79.5)
Antiarrhythmic drugs	1479 (36.1)	1052 (33.1)	805 (36.8)	1857 (34.6)
Antiplatelet drugs	274 (6.7)	307 (9.7)	275 (12.6)	582 (10.8)
Lipid-lowering drugs	1670 (40.7)	1227 (38.6)	920 (42.1)	2147 (40.0)
NSAIDs	45 (1.1)	34 (1.1)	15 (0.7)	49 (0.9)
Other	898 (21.9)	487 (15.3)	355 (16.2)	842 (15.7)

*Includes depression, psoriasis, thyropathy, vertebral algic syndrome and osteoporosis.

CHD, coronary heart disease; CVA, cerebrovascular accident; GI, gastrointestinal; MI, myocardial infarction; NSAIDs, non-steroidal anti-inflammatory drugs; TIA, transient ischaemic attack; VKA, vitamin K antagonist.

**Table 2:** Healthcare system characteristics (main analysis set)

	Cohort A (switched from VKA to dabigatran)		Cohort B (newly initiated on dabigatran or a VKA)	
	Total (n=4100)	Dabigatran (n=3179)	VKA (n=2186)	Total (n=5365)
Specialty of the treating physician, n (%)				
Cardiologist	3230 (78.8)	2682 (84.4)	1935 (88.5)	4617 (86.1)
General practitioner	96 (2.3)	82 (2.6)	27 (1.2)	109 (2.0)
Internist	607 (14.8)	202 (6.4)	90 (4.1)	292 (5.4)
Neurologist	153 (3.7)	198 (6.2)	126 (5.8)	324 (6.0)
Other	14 (0.3)	15 (0.5)	8 (0.4)	23 (0.4)
Healthcare reimbursement status, n (%)				
Partially reimbursed	490 (12.0)	26 (10.3)	342 (15.6)	668 (12.5)
Private pay	1430 (34.9)	1614 (50.8)	1018 (46.6)	2632 (49.1)
Reimbursed	2153 (52.5)	1197 (37.7)	808 (37.0)	2005 (37.4)
Other	27 (0.7)	42 (1.3)	18 (0.8)	60 (1.1)

VKA, vitamin K antagonist.

**Table 3:** Physician-rated risk scores (main analysis set)

	Cohort A (switched from VKA to dabigatran)		Cohort B (newly initiated on dabigatran or a VKA)	
	Total (n=4100)	Dabigatran (n=3179)	VKA (n=2186)	Total (n=5365)
CHA_2_DS_2_-VASc score (category), n (%)				
High risk (≥2)	3619 (88.3)	2791 (87.8)	1998 (91.4)	4789 (89.3)
Low/intermediate risk (<2)	222 (5.4)	262 (8.2)	153 (7.0)	415 (7.7)
Not available	259 (6.3)	126 (4.0)	35 (1.6)	161 (3.0)
CHA_2_DS_2_-VASc score (result), n (%)				
0	13 (0.3)	19 (0.6)	14 (0.6)	33 (0.6)
1	209 (5.1)	243 (7.6)	139 (6.4)	382 (7.1)
2	529 (12.9)	599 (18.8)	400 (18.3)	999 (18.6)
3	944 (23.0)	759 (23.9)	536 (24.5)	1295 (24.1)
4	875 (21.3)	643 (20.2)	481 (22.0)	1124 (21.0)
5	640 (15.6)	404 (12.7)	330 (15.1)	734 (13.7)
6	362 (8.8)	263 (8.3)	150 (6.9)	413 (7.7)
7	195 (4.8)	85 (2.7)	77 (3.5)	162 (3.0)
8	62 (1.5)	25 (0.8)	18 (0.8)	43 (0.8)
9	12 (0.3)	13 (0.4)	6 (0.3)	19 (0.4)
Not available	259 (6.3)	126 (4.0)	35 (1.6)	161 (3.0)
HAS-BLED score (category), n (%)				
High risk (≥3)	2429 (59.2)	925 (29.1)	685 (31.3)	1610 (30.0)
Low risk (<3)	1272 (31.0)	2039 (64.1)	1437 (65.7)	3476 (64.8)
Not available	399 (9.7)	215 (6.8)	64 (2.9)	279 (5.2)
HAS-BLED score (result), n (%)				
0	52 (1.3)	166 (5.2)	112 (5.1)	278 (5.2)
1	386 (9.4)	785 (24.7)	553 (25.3)	1338 (24.9)
2	834 (20.3)	1088 (34.2)	772 (35.3)	1860 (34.7)
3	1149 (28.0)	634 (19.9)	492 (22.5)	1126 (21.0)
4	865 (21.1)	223 (7.0)	150 (6.9)	373 (7.0)
5	313 (7.6)	61 (1.9)	39 (1.8)	100 (1.9)
6	82 (2.0)	6 (0.2)	4 (0.2)	10 (0.2)
7	18 (0.4)	1 (0.0)	–	1 (0.0)
8	2 (0.0)	–	–	–
Not available	399 (9.7)	215 (6.8)	64 (2.9)	279 (5.2)

VKA, vitamin K antagonist.

In both cohorts, most patients had comorbidities and were receiving concomitant medications: 86% of patients in cohorts A and B, and 83% and 91% of dabigatran and VKA-treated patients, respectively. The most frequently prescribed concomitant medication was antihypertensives, most of which were angiotensin-converting enzyme inhibitors (used by 1565 (38.2%) (cohort A) and 2119 (39.5%) (cohort B) patients). Most lipid-lowering agents were statins (used by 1620 (39.5%) (cohort A) and 2104 (39.2%) (cohort B) patients). Antiarrhythmic agents were mostly class III potassium channel blockers (used by 474 (11.6%) (cohort A) and 688 (12.8%) (cohort B) patients), although beta-adrenergic receptor blockers were used as antiarrhythmics in 736 (18.0%) patients in cohort A and 954 (17.8%) in cohort B. The most frequently prescribed antacid drugs were proton pump inhibitors, used by 360 (8.8%) patients in cohort A and 393 (7.3%) in cohort B; for antiplatelet drugs, it was acetylsalicylic acid, used by 218 (5.3%) patients in cohort A and 488 (9.1%) in cohort B.

Most cohort A (65%) patients and the dabigatran subgroup of cohort B (70%) were treated with dabigatran 150 mg two times per day; the remainder were treated with dabigatran 110 mg two times per day. In cohort A, the mean duration of previous VKA therapy was 34 months (median 19 months), with warfarin (n=2680 (65%)) and acenocoumarol (n=1233 (30%)) the most frequently used VKAs.

### Treatment perceptions (outcomes)

#### Primary outcome in patients switched from VKA to dabigatran (cohort A)

**Figure 1:**
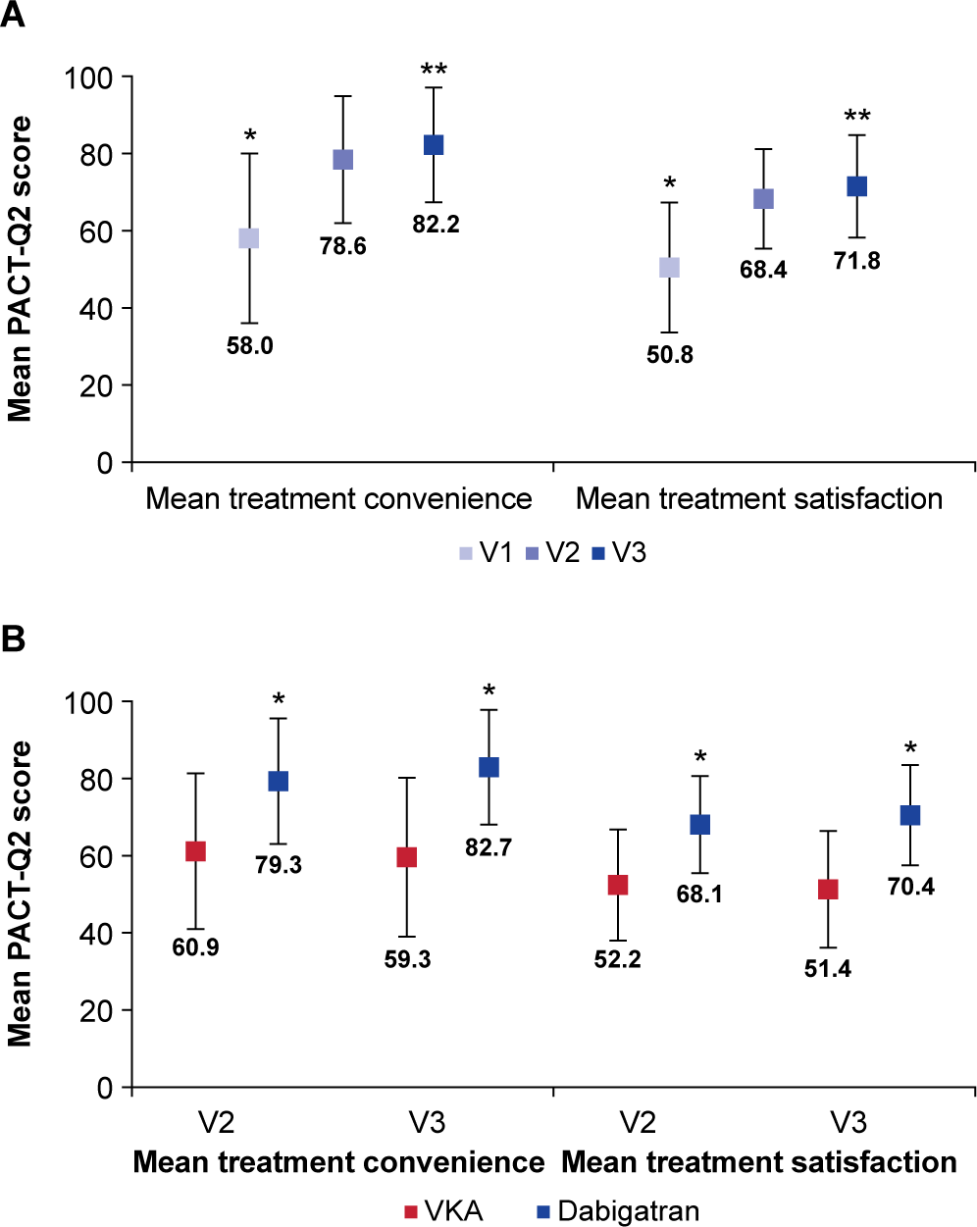
Mean PACT-Q2 treatment convenience and satisfaction scores. (A) Cohort A (patients switched from VKA to dabigatran). *P<0.001 V1 versus V2 and V1 versus V3. **P<0.001 V2 versus V3. Error bars represent SD. (B) Cohort B (patients newly initiated on dabigatran or a VKA). *P<0.001 dabigatran versus VKA. Error bars represent SD. PACT-Q, Perception on Anticoagulant Treatment Questionnaire; V1, baseline; V2, initiation period; V3, continuation period; VKA, vitamin K antagonist.

Mean PACT-Q2 treatment convenience and satisfaction scores are shown in figure 1A, with both scores improving significantly from baseline to V2 and V3. For treatment convenience, mean PACT-Q2 change from V1 at V2 was 20.72 (SD 21.50; p<0.001), and from V1 at V3 it was 24.54 (SD 22.85; p<0.001). For treatment satisfaction, mean PACT-Q2 satisfaction change from V1 at V2 was 17.60 (SD 18.76; p<0.001), and from V1 at V3 it was 21.04 (SD 20.24; p<0.001).

#### Secondary outcome in patients switched from VKA to dabigatran (cohort A)

The mean changes (improvements) over time in PACT-Q2 treatment convenience and satisfaction scores between V3 and V2 were also statistically significant (figure 1A). Mean change in PACT-Q2 scores between V3 and V2 for treatment convenience was 3.62 (SD 12.98; p<0.001), and for treatment satisfaction it was 3.33 (SD 12.86; p<0.001).

#### Primary outcome in patients newly initiated on dabigatran or a VKA (cohort B)

Mean PACT-Q2 scores for treatment convenience and satisfaction were significantly higher in the dabigatran group compared with the VKA group at both V2 and V3 (p<0.001 for all), with treatment differences increasing over time (figure 1B). For dabigatran versus VKA, mean treatment difference in PACT-Q2 scores for treatment convenience at V2 was 18.38 (SE 0.51), and at V3 it was 23.34 (SE 0.51). For treatment satisfaction at V2, it was 15.88 (SE 0.39), and at V3 it was 19.01 (SE 0.41). All differences in treatment convenience and satisfaction PACT-Q2 scores between treatment groups at both V2 and V3 were statistically significant (p<0.001).

#### Secondary outcome in patients newly initiated on dabigatran or a VKA (cohort B)

Table 4 summarises patient treatment expectations (PACT-Q1 items) at baseline in cohort B. Most patients were confident that their anticoagulant treatment would prevent blood clots (59% reporting ‘a lot’ or ‘extremely’) and had moderate-to-high expectation of symptom relief (65% reporting ‘moderately’, ‘a lot’ or ‘extremely’). The majority of patients considered it important to have an anticoagulant treatment that was easy to take (77% reporting ‘a lot’ or ‘extremely’).

**Table 4:** Description of treatment expectations by the patients (PACT-Q1 items) in cohort B (patients newly initiated on dabigatran or a VKA) in the main analysis set

PACT-Q1 item	Missing	Not at all	A little	Moderately	A lot	Extremely
A1—How confident are you that your anticoagulant treatment will prevent blood clots?						
Dabigatran*	103 (3.2)	46 (1.4)	230 (7.2)	803 (25.3)	1480 (46.6)	517 (16.3)
VKA†	64 (2.9)	32 (1.5)	215 (9.8)	701 (32.1)	946 (43.3)	228 (10.4)
Overall‡	167 (3.1)	78 (1.5)	445 (8.3)	1504 (28.0)	2426 (45.2)	745 (13.9)
A2—Do you expect that your anticoagulant treatment will relieve some of the symptoms you experience?						
Dabigatran*	103 (3.2)	361 (11.4)	607 (19.1)	944 (29.7)	911 (28.7)	253 (8.0)
VKA†	64 (2.9)	261 (11.9)	488 (22.3)	670 (30.6)	599 (27.4)	104 (4.8)
Overall‡	167 (3.1)	622 (11.6)	1095 (20.4)	1614 (30.1)	1510 (28.1)	357 (6.7)
A3—Do you expect that your anticoagulant treatment will cause side effects such as minor bruises or bleeding?						
Dabigatran*	103 (3.2)	487 (15.3)	1201 (37.8)	1012 (31.8)	320 (10.1)	56 (1.8)
VKA†	64 (2.9)	250 (11.4)	812 (37.1)	772 (35.3)	253 (11.6)	35 (1.6)
Overall‡	167 (3.1)	737 (13.7)	2013 (37.5)	1784 (33.3)	573 (10.7)	91 (1.7)
A4—How important is it for you to have an anticoagulant treatment that is easy to take?						
Dabigatran*	103 (3.2)	63 (2.0)	106 (3.3)	333 (10.5)	1676 (52.7)	898 (28.2)
VKA†	64 (2.9)	32 (1.5)	123 (5.6)	417 (19.1)	1153 (52.7)	397 (18.2)
Overall‡	167 (3.1)	95 (1.8)	229 (4.3)	750 (14.0)	2829 (52.7)	1295 (24.1)
A5—How concerned are you about making mistakes when taking your anticoagulant treatment?						
Dabigatran*	103 (3.2)	575 (18.1)	698 (22.0)	767 (24.1)	804 (25.3)	232 (7.3)
VKA†	64 (2.9)	208 (9.5)	491 (22.5)	646 (29.6)	607 (27.8)	170 (7.8)
Overall‡	167 (3.1)	783 (14.6)	1189 (22.2)	1413 (26.3)	1411 (26.3)	402 (7.5)
A6—How important is it for you to take care of your anticoagulant treatment by yourself?						
Dabigatran*	103 (3.2)	68 (2.1)	157 (4.9)	398 (12.5)	1599 (50.3)	854 (26.9)
VKA†	64 (2.9)	48 (2.2)	139 (6.4)	456 (20.9)	1097 (50.2)	382 (17.5)
Overall‡	167 (3.1)	116 (2.2)	296 (5.5)	854 (15.9)	2696 (50.3)	1236 (23.0)
A7—How concerned are you about how much you may have to pay for your anticoagulant treatment?						
Dabigatran*	103 (3.2)	398 (12.5)	495 (15.6)	1057 (33.2)	780 (24.5)	346 (10.9)
VKA†	64 (2.9)	183 (8.4)	186 (8.5)	396 (18.1)	824 (37.7)	533 (24.4)
Overall‡	167 (3.1)	581 (10.8)	681 (12.7)	1453 (27.1)	1604 (29.9)	879 (16.4)

All data are n (%).

*Dabigatran: n=3179.

†VKA: n=2186.

‡Total: n=5365.

PACT-Q, Perception on Anticoagulant Treatment Questionnaire; VKA, vitamin K antagonist.

### Safety outcomes

Similar numbers of ADRs or serious ADRs were reported by patients in cohorts A and B, and the dabigatran and VKA cohort B treatment groups (table 5). There were few severe ADRs: 12 (0.3%) patients in cohort A and 21 (0.4%) in cohort B (dabigatran: 14 (0.4%); VKA: 7 (0.3%)). ADRs leading to discontinuation were reported in 43 (1.1%) patients in cohort A and 36 (0.7%) in cohort B (dabigatran: 32 (1.0%); VKA: 4 (0.2%)). At the system organ class level, the most frequently reported ADRs leading to discontinuation were gastrointestinal (GI) disorders (the most common being abdominal pain, dyspepsia and GI haemorrhage), reported in 24 (0.6%) patients in cohort A and 17 (0.3%) in cohort B (dabigatran: 15 (0.5%); VKA: 2 (0.1%)). Nine patients experienced ischaemic stroke: three (0.1%) in cohort A, six (0.1%) in cohort B (dabigatran: 5 (0.2%); VKA: 1 (0.0%)); of these, two cohort A patients discontinued and one in the cohort B VKA group died. Few patients experienced fatal ADRs: five (0.1%) in cohort A and six (0.1%) in cohort B (dabigatran: 2 (0.1%); VKA: 4 (0.2%)).

**Table 5:** Summary of ADRs and observed person-years (ie, time at risk) (safety analysis set)

	Cohort A (switched from VKA to dabigatran)						Cohort B (newly initiated on dabigatran or a VKA)					
	Total (n=4066)			Dabigatran (n=3164)			VKA (n=2181)			Total (n=5345)		
	n (%)	Time at risk, pt-yrs	Rate/ 100 pt-yrs	n (%)	Time at risk, pt-yrs	Rate/ 100 pt-yrs	n (%)	Time at risk, pt-yrs	Rate/ 100 pt-yrs	n (%)	Time at risk, pt-yrs	Rate/ 100 pt-yrs
Any ADRs	94 (2.3)	1967	4.78	80 (2.5)	1532	5.22	60 (2.8)	1073	5.59	140 (2.6)	2605	5.37
Any bleeding ADR	27 (0.7)	NE	NE	30 (0.9)	NE	NE	39 (1.8)	NE	NE	69 (1.3)	NE	NE
Serious ADRs	26 (0.6)	1981	1.31	25 (0.8)	1541	1.62	15 (0.7)	1084	1.38	40 (0.7)	2625	1.52
Serious bleeding ADR	8 (0.2)	NE	NE	4 (0.1)	NE	NE	3 (0.1)	NE	NE	7 (0.1)	NE	NE
ADRs leading to discontinuation	43 (1.1)	1981	2.17	32 (1.0)	1544	2.07	4 (0.2)	1086	0.37	36 (0.7)	2630	1.37
Bleeding ADRs leading to discontinuation	13 (0.3)	NE	NE	16 (0.5)	NE	NE	2 (0.1)	NE	NE	18 (0.3)	NE	NE
Fatal ADRs	5 (0.1)	1984	0.25	2 (0.1)	1545	0.13	4 (0.2)	1086	0.37	6 (0.1)	2631	0.23
Fatal bleeding ADR	0	NE	NE	0	NE	NE	0	NE	NE	0	NE	NE

ADR, adverse drug reaction; NE, not evaluated; pt-yrs, patient-years; VKA, vitamin K antagonist.

The number of bleeding ADRs was small (table 5), with the most common being GI and gingival bleeding events. Four severe bleeding ADRs (GI, tumour, haemorrhoidal and cerebral) were reported in four (0.1%) patients in cohort B (dabigatran: 3 (0.1%); VKA: 1 (0.0%)). Bleeding ADRs leading to discontinuation were reported by 13 (0.3%) patients in cohort A and 20 (0.4%) in cohort B (dabigatran: 18 (0.6%); VKA: 2 (0.1%)). No patients experienced a fatal bleeding ADR.

## Discussion

There are limited data on patients’ perceptions of long-term anticoagulation therapy in NVAF. In this observational study, which was a representative population of 9472 patients with NVAF in 11 European countries and Israel, treatment satisfaction and convenience in patients who switched from a VKA to dabigatran increased significantly from baseline over time. For those patients newly diagnosed with NVAF, treatment satisfaction and convenience were significantly higher for dabigatran compared with VKA therapy. Few, serious and severe ADRs were reported by similar numbers of patients in cohorts A and B, and in dabigatran-treated and VKA-treated cohort B patients. While numbers were low, patients receiving dabigatran versus a VKA reported more ADRs that led to treatment discontinuation, with GI events driving approximately half the discontinuations. However, no difference was observed in severe bleeding events in patients receiving dabigatran compared with VKA. Overall, the safety profile of dabigatran in the RE-SONANCE study was consistent with previous clinical dabigatran and VKA data in the AF setting.^15–17^

Few studies have assessed patients’ perspectives of anticoagulant therapy in AF. In contrast to our findings, a substudy of the RE-LY trial observed stable health-related quality of life (measured using EQ-5D) scores over 12 months in all treated patients without outcome events, with comparable scores in the dabigatran and warfarin groups.^13^ This was unexpected, given the known complexities of warfarin treatment. In the PREFER in AF registry, within the first year of NOACs being available in Western Europe, patient-related factors influencing the switch from a VKA to a NOAC included complaints about bruising/bleeding, treatment dissatisfaction, mobility problems and anxiety/depression.^11^ Patients switching from a VKA to a NOAC had less hypertension, heart valve dysfunction and CHA_2_DS_2_-VASc scores,^11^ possibly reflecting caution on the part of physicians trying a new treatment option. In a small study using the validated Anti-Clot Treatment Scale, warfarin treatment was less favoured than non-warfarin treatments, including more limitations and greater feelings of burden.^18^

It is recognised that there is a need for improvement in educational strategies around OACs^6 9^; in order to anticoagulate patients successfully with AF at high risk for stroke and prevent further ischaemic stroke, physicians and patients need to understand fully the rationale behind OAC treatment. Physicians must be provided with evidence regarding which treatment option best suits their patients’ clinical presentation. For example, NOACs should be the first option physicians consider for preventing stroke in patients with AF (including aortic regurgitation or stenosis), although VKAs are indicated for preventing stroke in those patients with AF and a mechanical valve or moderate-to-severe mitral valve stenosis.^3^ For patients, providing educational strategies can significantly improve their OAC treatment knowledge compared with usual care.^8^ By regularly evaluating knowledge gaps (eg, international normalised ratio target ranges, which concomitant medications should be avoided and recognising medical complications such as stroke or bleeding), the most appropriate educational programmes can be provided. Such strategies have been shown to increase patients’ use of OACs significantly^5^ and the number of patients achieving time within therapeutic range.^7^ Furthermore, educational strategies can significantly reduce the risk of recurrent stroke compared with usual care.^5^

Other studies have observed higher treatment satisfaction among NOAC versus warfarin users.^19–22^ It is reasonable to extrapolate that improving anticoagulation treatment satisfaction may increase patients’ adherence to treatment and improve outcomes. Open discussions with patients can help to identify potential barriers to therapy; if patients are involved in their treatment decisions, they may be more likely to take responsibility for their treatment, thereby improving adherence.^23^ However, despite improved treatment satisfaction with NOACs versus VKAs, recent observational data noted comparable adherence.^21 22^ The paucity of data regarding treatment satisfaction and outcomes means that further studies are needed to assess the impact of improved patient perceptions.

A strength of this study is that it used the PACT-Q, which is a validated and specific treatment satisfaction instrument for thromboembolic patients with anticoagulant treatment, and is available in numerous languages.^10 14^ While there are inherent limitations associated with any observational study, the large patient population recruited in this non-interventional study is representative of patients receiving an OAC for stroke prevention in NVAF. Study limitations include the role of reimbursement, which could affect overall treatment adherence and satisfaction, and is highly dependent on a patient’s financial and socioeconomic status. Patient selection bias may also be the reason why only one-third of patients were over 75 years; elderly patients, who could benefit from switching from VKA to dabigatran, may not have been included due to the perception that they might not be able to understand the questionnaires fully. Treatment bias may also have been introduced, as patients might subconsciously consider a new therapy as better. Additionally, due to the real-world nature of the study, patient follow-up was based on routine care instead of a stringent visit schedule, as used in clinical trials. Therefore, there was considerable variation between patient visits from baseline during the study follow-up. To better accommodate this and ensure that most of the collected data could be included in the analysis, more relaxed time windows were defined for V2 and V3. The balance between time window thresholds and potential misclassification was assessed, and it was decided to apply ‘consecutive’ thresholds between V2 and V3 to capture as much of the data as possible, and since the number of patients with extreme visit times was low. Finally, patients may not always be willing to write their honest opinions on a questionnaire or tell the doctor what they really think about the therapy. Although not aimed at assessing patient outcome, the safety profile of the OACs were comparable.

## Conclusions

Switching to dabigatran from long-term VKA therapy or newly initiated dabigatran is associated with improved patient treatment convenience and satisfaction compared with VKA therapy. This may be important in the prevention of stroke and systemic embolism in patients with AF at moderate-to-high risk.
