# Supplementary figures and images for: Patient perception of anticoagulant treatment for stroke prevention (RE-SONANCE study)
Source: Open Heart. 2020 Mar 24;7(1):e001202. doi: 10.1136/openhrt-2019-001202 (PMC7103803; doi:10.1136/openhrt-2019-001202)

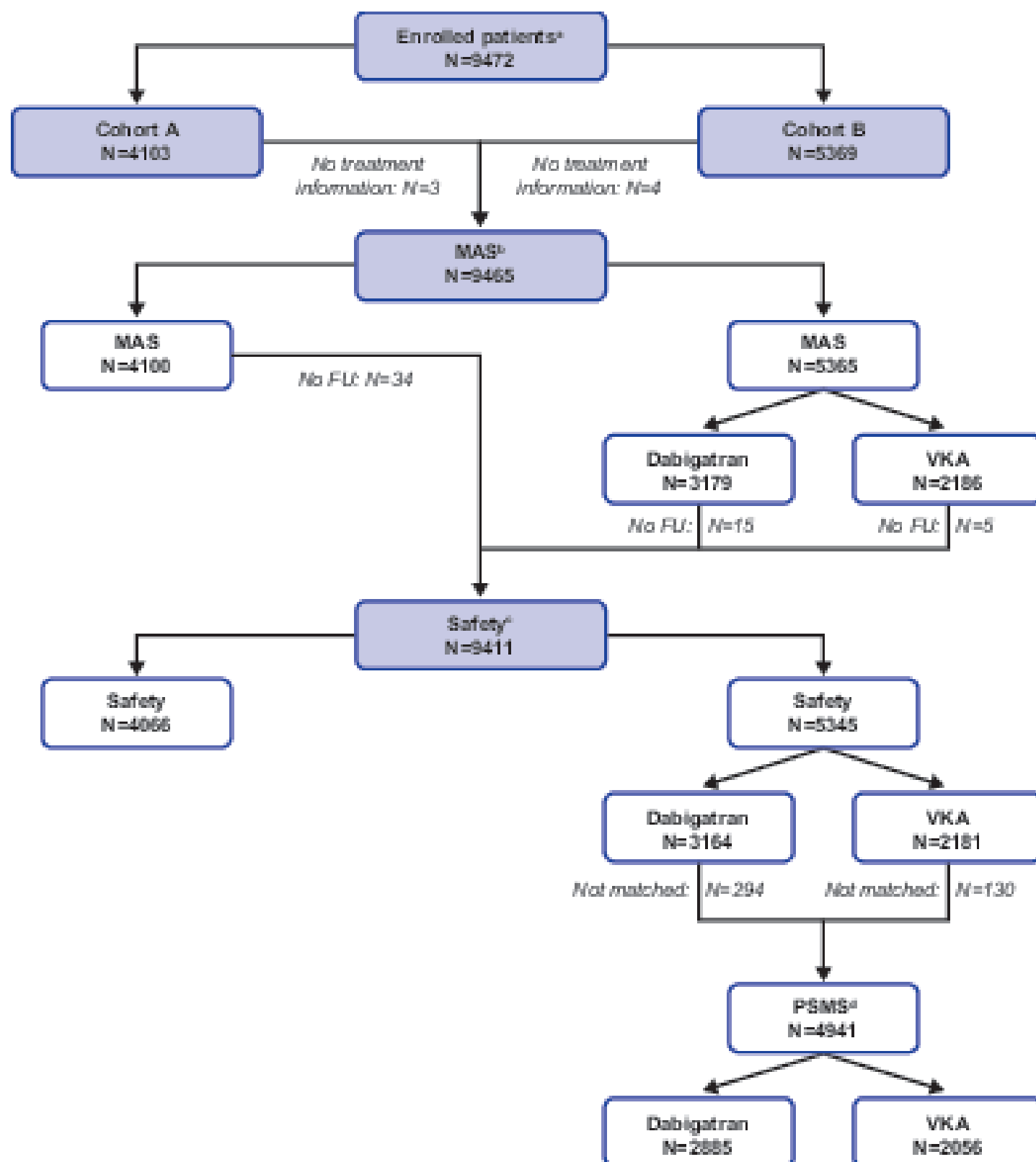

Supplement: Supplementary data [file openhrt-2019-001202supp001.pdf]
